# Evaluating a tool to improve engagement and recruitment of under-served groups in trials

**DOI:** 10.1186/s13063-022-06747-2

**Published:** 2022-10-09

**Authors:** Lydia Morris, Jo Dumville, Shaun Treweek, Nasima Miah, Ffion Curtis, Peter Bower

**Affiliations:** 1 NIHR Applied Research Collaboration Greater Manchester, Manchester Academic Health Science Centre, Research and Innovation Division, Manchester University Foundation NHS Trust, Manchester, UK; 2 Division of Psychology and Mental Health, School of Health Sciences, Faculty of Biology, Medicine & Health, University of Manchester, Manchester, UK; 3 Division of Nursing, Midwifery & Social Work, School of Health Sciences, Faculty of Biology, Medicine & Health, University of Manchester, Manchester, UK; 4 Health Services Research Unit, University of Aberdeen, Aberdeen, UK; 5 The Centre for Ethnic Health Research, NIHR ARC East Midlands, Nottingham, UK; 6 Division of Population Health, Health Services Research & Primary Care, School of Health Sciences, Faculty of Biology, Medicine & Health, University of Manchester, Manchester, UK

**Keywords:** Trial methodology, Inclusion under-served groups, Ethnic minority groups, Patient, public involvement (PPI), Patient, public and community involvement and engagement (PCIE), Minoritised groups

## Abstract

**Background:**

Despite substantial awareness that certain groups (e.g. ethnic minorities) are under-represented and under-served in trials, limited progress has been made in addressing this. As well as a public service and ethical duty to recruit and engage under-served groups in relevant research, importantly, there are clear scientific benefits, for example, increased generalisability.

The key aims of the current study were to explore the following: general barriers and facilitators to enhancing the recruitment of under-served groups into trials, the usability and value of a specific tool (INCLUDE Ethnicity Framework) to support engagement and recruitment of under-served groups, and ways of engaging diverse patient, public and community involvement and engagement (PCIE) groups.

**Methods:**

Firstly, researchers completed a brief survey in relation to a specific trial in which they were involved (*N* = 182, 38% response rate). A second stage involved sampling survey respondents and asking them to complete the INCLUDE Ethnicity Framework and then a remote semi-structured interview (*N* = 15). Qualitative data were analysed using thematic analysis. Finally, we conducted a consultation process with PCIE contributors primarily to develop guidelines for discussing the INCLUDE Ethnicity Framework with PCIE representatives.

**Results:**

Researchers recognised the importance of increasing engagement and recruitment of under-served groups within trials, but varied in their knowledge, ability and commitment to implementation in practice. The INCLUDE Ethnicity Framework was described by some as raising their awareness of how inclusion could be improved. Respondents highlighted a need for shared resources and wider structural change to facilitate such engagement. PCIE was identified, in the survey and interviews, as the most common method of trying to improve recruitment of under-served groups. However, researchers also commonly highlighted that PCIE groups were sometimes not very diverse.

**Conclusions:**

There is a need for researchers to consider the funding and time resources required for diverse and inclusive recruitment to trials and for funders to enable this. The INCLUDE Ethnicity Framework can help to raise awareness of inclusion challenges. This study indicates that it is important to take proactive steps to involve relevant under-served groups in PCIE and practical suggestions are made to facilitate this.

**Supplementary Information:**

The online version contains supplementary material available at 10.1186/s13063-022-06747-2.

## Introduction

Despite substantial awareness that certain groups (e.g. ethnic minorities) are under-served in research (e.g. [3, 4, 18]), limited progress has been made in addressing this (e.g. [4, 18]). A recent rapid review of the literature highlighted the multifaceted barriers to inclusion, as well as identifying a range of strategies that could address some of these barriers [1].

A UK National Institute for Health Research (NIHR) Clinical Research Network-funded programme of research (Innovations in Clinical Trial Design and Delivery for the Under-served; INCLUDE) identified that the term ‘under-served groups’ was preferred by a range of stakeholders, including a range of representatives from such groups [18]. This term applies to those groups sometimes described as ‘under-represented’, ‘hard to reach’ or ‘seldom heard’. Some key characteristics of under-served groups are as follows: lower inclusion in research than population estimates suggest is needed, high healthcare burden that is not matched by the volume of research and important differences in how a group responds or engages to interventions, services or research [18].

Greater inclusion in health research is an ethical and equity imperative but it is also a scientific one. For example, it is important to ensure that trial results are generalisable to the relevant population and that important findings specific to different populations are not missed [18]. There can be treatment differences between different under-served groups; for example, a meta-analytic review found that ACE inhibitors for hypertension are less effective in African Americans [15], and there can be differences in experience and access to both psychological and healthcare interventions across different ethnic minority and socio-economic groups [10, 11].

As described in previous studies, there are inter-related reasons for the lack of diversity among research participants, such as how trials are designed, where they are delivered and a lack of trust in the research process in some communities [1, 9]. The INCLUDE programme generated new resources to facilitate engagement and recruitment of under-served groups specifically in randomised trials. One of the resources is the INCLUDE Ethnicity Framework, which asks a series of questions to encourage researchers developing trials to consider inclusion in the design phase. The Framework has two components, the first on general inclusion and the second focusing on inclusion of different ethnic groups. Although designed collaboratively with researchers, funders and public contributors, experience with the Framework is limited, and it is important to understand this early experience of its use in the research community, which was the focus of this work. We also took the opportunity to explore the views of researchers on other approaches to stimulate inclusion of under-served groups in research, such as mandating including ethnic minority participants in line with population prevalence as used in The National Institutes of Health (NIH)-funded research in the USA.

The key aims of the current study were to explore general barriers and facilitators to enhancing the recruitment of under-served groups into trials, the usability and value of a specific tool (INCLUDE Ethnicity Framework) to support engagement and recruitment of under-served groups, and ways of engaging diverse patient, public and community involvement and engagement (PCIE) groups into research. The focus of the study was on recruitment of under-served groups but questions were also asked about the involvement and retention of individuals from such groups within trials.

Below are the questions we were addressing and the overall methods used:

- What barriers do research teams perceive to enhancing the recruitment of under-served groups into trials and what are the current approaches used? This was examined using a survey and a semi-structured online interview.
- What are researcher views on the usability and value of the INCLUDE Ethnicity Framework in supporting engagement and recruitment of under-served groups into trials? Examined via completion of the INCLUDE Ethnicity Framework and semi-structured online interview.
- Are there areas of future development and resourcing that may improve engagement and recruitment of people from under-served groups into research? Examined using a survey and a semi-structured online interview.
- What are the optimal ways of engaging diverse patient, public and community advisory groups (often described as PPI, PPIE or PCIE groups) and effectively enabling them to engage with the INCLUDE Ethnicity Framework? Examined via a consultation process with public contributor advisory group.

## Methods

In order to meet the aims, we combined three sources of data:

- Survey: a survey of researchers working on trials for NIHR, a UK funder
- Qualitative interviews with trialists: INCLUDE Ethnicity Frameworks were completed by a subsample of researchers identified via the survey, and then all these researchers completed a qualitative interview to explore perceptions of the Ethnicity Framework and its acceptability
- Consultation process with public contributor advisory group: feedback from multiple patient, public and community advisory groups across two locations on a brief guidance document for researchers regarding involving patient, public and community involvement and engagement (PCIE) contributors in the INCLUDE Ethnicity Framework

### Survey

We recruited researchers, initially targeting chief investigators (CIs), responsible for preparing, submitting or running trials for the NIHR Research for Patient Benefit (RfPB), Invention for Innovation (I4I), Efficacy and Mechanism Evaluation (EME) and Health Technology Assessment (HTA) funding streams (in the last 24 months; start dates March 2019–March 2021). The most relevant studies were expected to be within HTA funding as these are generally expected to be of immediate clinical relevance, but we included a range of potential NIHR funding streams because considering inclusion is important more widely within trials. We chose to focus on the NIHR as it is the major funder of applied health and care research in the UK.

We identified relevant CIs from NIHR databases and emailed them details of the survey. All CIs were asked to complete initial survey questions in relation to a specific trial that they had recently received, or applied for, NIHR funding. They provided consent to participate within the survey platform before being taken to the survey page. As part of returning their answers, they were asked to confirm whether they were willing to take part in qualitative interviews concerning the INCLUDE Ethnicity Framework.

We aimed to gather data pertaining to activity from at least 50 different CIs of studies from the last 24 months. Survey data were entered and stored in a secure database. Analysis was descriptive, largely summarising response data using proportions and measures of variance.

### Qualitative interviews with trialists

We sampled participants from consenting survey respondents. Unlike the survey (which targeted CIs), we specified that CIs could invite trial managers to complete the task and interview.

Potential participants were contacted with details of this sub-study. This involved researchers completing the INCLUDE Ethnicity Framework (either the general questions about a range of under-served populations or the specific questions about inclusion of different ethnic groups) in relation to a specific trial for which they had recently received, or applied for, NIHR funding. The first author then interviewed all researchers who completed an Ethnicity Framework.

For the completion of the INCLUDE Ethnicity Framework and qualitative interview, we used purposive sampling to ensure coverage of key intervention classes of interest: drug trials, device trials and complex intervention trials (physical interventions that are not device trials, and psychosocial interventions). The procedure regarding the INCLUDE Ethnicity Framework and the subsequent follow-up was piloted with a trial manager outside of the direct project team and adaptations were made to the procedure and interview schedule.

The interview element was a brief semi-structured interview via a video call that asked more detailed questions regarding the researcher’s experience of using the INCLUDE Ethnicity Framework. The interview schedule was developed by the study team (LM, JD, PB, ST; all of whom have experience of leading or managing trials). At the qualitative interview, LM explained the interview procedure, the rights of the participant (e.g. the ways in which information from the interview would be used) and encouraged questions. If the participant was willing to go ahead, they were consented.

The participants in this qualitative study were relatively homogenous (researchers working on trials). Our target sample size was 5–6 for the general questions at the beginning of the Ethnicity Framework (which can be used to consider a range of under-served groups). Our target sample size for the detailed worksheets that constitute the main ‘body’ of the Ethnicity Framework was 8–10. This was to make the task more manageable for researchers completing the INCLUDE Ethnicity Framework and to get an indication whether the worksheets enhanced usability. Thus, our overall sample size target was 13–16 interviews. All interviews were analysed together using thematic analysis and followed Braun and Clarke’s [2] six-phase approach within a critical realist framework [5].

### Consultation process with public contributor advisory group

The aim of the consultation process was to develop supporting material to engage diverse patient, public and community advisory groups when using the INCLUDE Ethnicity Framework. Such supporting material was intended to include resources to encourage researchers to draw on PCIE expertise in completing the INCLUDE Ethnicity Framework.

PCIE contributors were recruited through Greater Manchester and East Midlands NIHR Applied Research Collaborations.

Initially, the consultations were conducted via two separate groups that met in parallel, one from East Midlands and one from Greater Manchester. These two groups met separately initially (two to three meetings with the group from each area) and then merged (one meeting). The separate group sizes were five (Greater Manchester) and seven (East Midlands). The project involved monthly online PCIE meetings over a 4-month period with meetings lasting around an hour and a half.

The consultation process used an online focus group format designed to systematically capture and incorporate the suggestions and recommendations of the group [8]. Materials were generally sent out prior to the meetings as well as being reviewed during the groups. The screen share function was used to share the documents that were being co-created by the group, primarily this was a ‘Guidance for researchers to involve patient, public and community contributors in the INCLUDE Ethnicity Framework’ document.

## Results

### Participant flow and characteristics (see Additional file 1 for the participant flow diagram)

Eight-hundred and twenty-three studies were reviewed from NIHR funding databases and minutes. Of these, 485 were potentially eligible and had a named CI with a functioning email contact; 182 out of 485 responded (38%).

At the end of the survey, 110 CIs (60%) consented to be contacted about completing an INCLUDE Ethnicity Framework and being interviewed about this, or nominated their trial manager to complete the Ethnicity Framework and interview. Fifteen CIs or trial managers completed a Framework and interview.

## Survey findings (see Table 1)

In total, 115/182 (63.2%) respondents reported under-served groups as being relevant to their trial. In response to the question ‘Has the design or conduct of the trial been informed by members of the under-served population identified as important?’, 85.1% of CIs (97/114) stated ‘Yes’. A range of approaches to the involvement of members of the under-served groups were reported (Table 1) with the most common (88/115; 76.5%) being ‘Patient and Public Involvement (PPI) representatives from under-served groups will be/are advisors’. Furthermore, the most common approach that was considered by trial teams to increase the recruitment of under-served groups was PPI (81.6%; 102/125).

**Table 1:** Summary of key survey results

Question ***n*** = answered within this section	Response option	% (***n***)
Under-served groups that researchers identified as important for their particular trial *n* = 115	Ethnic minorities	44.3% (51)
	LGBTQ+	7.0% (8)
	People with cognitive impairments	22.6% (26)
	Socio-economic disadvantage/low-income	53.9% (62)
	Male/female gender (depending on context)	20.9% (24)
	Age extremes (e.g. under 18 and over 75)	40.9% (47)
	People living in remote areas	20.9% (24)
	Religious minorities	10.4% (12)
	Other (e.g. people with physical disabilities; with complex or severe mental health needs; substance users; carers)	26.1% (30)
The previous/planned involvement of members of this under-served population within the relevant trial *n* = 115	Review of funding application	40.0% (46)
	Patient and public involvement (PPI) representatives from under-served groups will be/are advisors	76.5% (88)
	PPI/service user researcher(s) from under-served population	36.5% (42)
	PPI from under-served groups to co-create intervention or other aspects of study design	41.7% (48)
	None	3.5% (4)
	Other (e.g. recruiting more participants from under-served populations into the trial)	11.3% (13)
How researchers identified the under-served groups that were relevant to their trial *n* = 115	Previous experience	85.2% (98)
	Research literature	35.7% (41)
	Toolkit or set of guidelines (e.g. INVOLVE and PROGRESS-Plus)	1.7% (2)
	Other (e.g. review of the clinical epidemiology of the target illness; support groups; PPI)	17.4% (20)
Approaches to increasing recruitment of under-served groups considered by trial teams *n* = 125	Patient and public involvement	81.6% (102)
	Staff training	43.2% (54)
	Recruiting from community organisations	32.8% (41)
	Cultural adaptations	23.2% (29)
	Use of toolkit to identify under-served groups	8.8% (11)
	Other (e.g. design of recruitment materials; recruiting from deprived areas)	23.2% (29)
Researcher views on funders mandating recruitment and inclusion of under-served groups *n* = 173	It would be difficult to have a quota for all groups	61.8% (107)
	Having a quota is a good idea	8.7% (15)
	Funding would be required to increase inclusion	45.1% (78)
	Mandating inclusion is not relevant to all trials	54.3% (94)
	Other	4.6% (8)

Researcher views on funders mandating recruitment and inclusion of under-served groups (e.g. via a quota) were mixed, but only 8.7% (15/173) stated that ‘Having a quota is a good idea’. Other perspectives were as follows: ‘It would be difficult to have a quota for all groups’ (61.8%; *n* = 107), ‘Funding would be required to increase inclusion’ (45.1%; *n* = 78), and ‘Mandating inclusion is not relevant to all trials’ (54.3%; *n* = 94).

## Qualitative findings

Primarily, these data reflect the qualitative interviews. However, both during the interviews and within the analysis process, references are made to the INCLUDE Ethnicity Framework, which were completed by all researchers who completed a qualitative interview. All researchers completed an aspect of the Ethnicity Framework and the qualitative interviews asked them about their experience of doing this, as well as related questions regarding their experience of recruiting and involving under-served groups in trials. Table 2 details the themes and subthemes.

**Table 2:** Superordinate and subordinate themes

Superordinate theme	Subordinate theme
Current barriers and strategies	Structural barriers
	Researcher barriers
	Current strategies
Enhancing engagement and recruitment of under-served groups	Resources suggested
	Structural changes
Usability and value of the framework	Usefulness of the Framework and general impressions
	Barriers to implementing the framework
	Usability issues with the Framework

## Current barriers and strategies (to increasing engagement and recruitment of under-served groups in trials)

### Structural barriers

Several structural barriers were identified by researchers, such as inequalities within service access or specific issues related to trials that were laboratory based or had to recruit a very specific population.

“So I suppose the barrier first of all is if people aren’t accessing those services, then they may not access the trial.” ID12

“So people from deprived and minority ethnic groups will struggle to get access to services in the first place. There’s quite a lot of hesitancy around medical interventions for mental health disorders themselves and we’re basically asking people to take an additional medication.” ID15

These were commonly described as being especially difficult for researchers to address:

“One of our issues is that the pool we are recruiting from is already not representative and how you fix all those barriers to women’s health and accessing care.” ID11

Also, time was seen as a barrier because measures to increase recruitment of under-served groups were identified by researchers as likely to increase study timelines (either based on their previous experience of this or based on the likely impact of potential strategies they were considering). Related to this was the (potential) impact on recruitment rates if recruiting from under-served sites that are not commonly involved in research.

“I think that they’ll (new, diverse sites) be hard to recruit in because they’re not as research active… We really need to persist with them, with those sites, because we’ll get very different participants. And also acknowledging that I think it will make it harder.” ID12

Another barrier commonly identified was funder awareness and acceptance of the need to engage and recruit more under-served groups. Researchers described mixed experiences regarding the extent to which the changes that funders and regulatory bodies required (from a range of funding bodies) considered the impact on equality and diversity. For example, requiring a pregnancy test and ongoing use of two types of contraception for participants in a trial of a particular medication, even though this medication is routinely used in pregnancy and within young women of childbearing age. This is clearly going to make it more challenging to recruit women because participation adds additional burden and excludes individuals who may be trying to conceive.

### Researcher barriers

The most common researcher barrier was lack of awareness of which under-served groups they should be involving and how to involve them. This was not always stated directly but sometimes could be inferred by questions to the interviewer or by statements that indicated that the wider context had not been considered. For example, in the statement below, the researcher is not aware of specific cultural groups who may have greater distrust in research for historical and other reasons.

“It would be difficult to know in advance which ones [ethnic groups] might be more or less receptive to research, and, I mean, I don’t know which cultures those might be.” ID7

How to engage and recruit under-served groups is not a straightforward question and cultural groups are not homogenous. However, some researchers described an increased awareness of barriers to engagement of under-served groups through completing the INCLUDE Ethnicity Framework.

“It got me thinking about the people who we actually don’t include into the study…. It’s various factors, like not having the study documentation in different languages.” ID10

A few researchers had already thought about under-served groups and the barriers they might face in some detail prior to completing the INCLUDE Ethnicity Framework and had implemented potential strategies to address these. This indicates that with some awareness of barriers and of what might help, some researchers were potentially able to implement ways of trying to engage and recruit under-served groups.

### Current strategies

Many researchers described one or two strategies that they were using to involve under-served groups. Public, patient and community involvement (PCIE or PPI) via advisory groups and individuals was one of the most commonly described strategies for engagement:

“This [the Ethnicity Framework] would be so helpful to have during the design phase and then we could appoint our PPI representatives more appropriately perhaps.”

Nearly all of the researchers who described using PCIE also described concerns regarding the diversity of the groups:

“It’s mainly white middle-class at the moment…it’s a challenge to open it out, it’s easier with ethnic minority groups… but I think it’s more difficult with people from socioeconomic deprived backgrounds to get representation in the PPI group from those groups.” ID13

“Most of our PPI groups all across the country are not necessarily representative of the population.” ID1

“I really don’t want it to be an exclusively white middle class university educated panel.” ID11

Other strategies described and used by several people included the use of translators. However, additional issues were identified regarding standardised and validated measures and whether translation would invalidate such measures:

“Those (questionnaires) are all standardised so it’s not like we could get them translated easily.” ID8

Site selection was mentioned as a way of improving inclusion (mainly regarding ethnic minorities but also sometimes considering socio-economic factors):

“We are recruiting from clinical teams with high numbers of Black and South Asian patient populations, in areas of economic deprivation.” ID15

## Enhancing engagement and recruitment of under-served groups

### Resources suggested

Researchers identified a range of resources that they would find helpful to increase engagement and recruitment of under-served groups. These resources included:

“Some central mechanism for translation and production of patient information sheets that was in multiple languages, that would have been useful.” ID7

Other ideas were someone to support with engagement and recruitment of under-served groups when preparing funding applications and the provision of guidance regarding translating validated measures:

“Whether the NIHR could provide someone to input into studies and whether there could be maybe someone that you could go to and have that point of contact.” ID10

Further suggestions included training for researchers, such as embedding training on this within pre-existing structures or institutional training packages.

### Structural changes

Researchers identified changes that would be useful for funders to implement. These included requiring that researchers consider and embed engagement and recruitment of under-served groups.

“I wondered if this was something that in phase two of the grant applications you should have a very abbreviated version of the form and then actually, I think it will impact on the trial design.” ID11

“I’m surprised that NIHR, for example, when then they’re… considering funding, don’t say, look… why haven’t you included budget for things like accessible formats, large print, and audio, and things like that for your patient facing materials.” ID8

It was also recognised that regulatory bodies could have a requirement to consider and embed engagement and recruitment of under-served groups.

“Even if it was part of some sort of IRAS [NHS ethics] application or something as well.” ID10

This was particularly identified as important for commercially funded trials where issues of representation were less likely to be considered than for publicly funded trials (e.g. Research Council and NIHR funded).

“Of course the regulatory system does have a means of influence in that. So for drug trials it would be the MHRA and the HRA… And the UK Medicines Agency. So they all now could be saying actually we want to show that diversity is present, so that you can be sure that the medicines will work in the full range of population.” ID3

## Usability and value of the framework

### Usefulness of the Framework and general impressions

Several participants commented on how useful they found the INCLUDE framework.

“I think it was really useful to actually complete, and it would have been really useful to have that at the beginning of every study so it does get that initial thought-provoking ideas and stuff in place.” ID10

“Overall I generally felt it was a very comprehensive questionnaire and it did what it was supposed to do, put it that way.” ID1

However, only a few identified that they had made changes to their trial as a result of using the framework.

“It’s a very useful, sort of, checklist to go through, to make sure that you're not making any obvious blunders when you set it up. So I think I found it helpful from that point. But, as I say, I'd struggle to say it was definitely this that we changed as a result of it.” ID3

This was partly due to the fact that some of the trials were recruiting, but also several identified that they found it difficult to turn the responses to the Framework into practical actions:

“Prompts would be good because the, the questions…I mean, the questions are very open ended.” ID8

Some researchers identified there should be more emphasis on the actions a researcher should take:

“(If) you answered the question and then there were some actions that you wanted to take, I think that would be really helpful…. There was a bit at the end after the worksheet that was actually about what you were going to do about it, wasn’t there…and I had other meetings and I just had to kind of leave that.” ID12

Or more emphasis on setting up a system within an individual trial to improve engagement and recruitment of under-served groups:

“But I think to expect the researchers to have the answers to those questions before doing the trial is quite a stretch. But I think they should set up the systems before the trial so that they can answer the questions before and during the trial.” ID2

### Barriers to implementing the framework

Barriers to completing and implementing the INCLUDE Ethnicity Framework, i.e. utilising strategies or making changes to the study design to improve engagement or recruitment of under-served groups, included the general barriers already detailed, such as inequalities within service access. However, there were other barriers that related more specifically to either completing or implementing the INCLUDE Ethnicity Framework. One of the most commonly mentioned barriers was researcher time to complete the Ethnicity Framework and consider fully the implications for different under-served groups (covered in more detail in the ‘Usability issues with the INCLUDE Ethnicity Framework’ section).

The most common barrier researchers identified was the time point that they were completing the INCLUDE Ethnicity Framework, because many of them had received their funding and some were recruiting. They identified that completing it when applying for funding would be helpful. Several researchers also identified that it was/would be useful when designing the protocol and creating study documents.

“It was very clear, and I think it’s a really good exercise if you’re at the point of designing your trial.” ID8

“If I, kind of, had this information when I was developing the documentation, it could have been a case of, actually, do we want to think of X, Y and Z to have this or…and when they were, kind of, developing the protocol for the study and obviously applying for the grant, thinking about those aspects and stuff as well, to try and include as many people as possible into the study.” ID14

### Usability issues with the INCLUDE Ethnicity Framework

One aim of the project was to identify any usability issues with the Framework so that they could be addressed in later versions.

Many researchers commented that they struggled with the length of the Ethnicity Framework and wondered if it could be made more concise. This was related to the issue of not having time to complete it, and several people said they gave up or did not consider the implications because it was too long.

“I did the first part, I was like, yay…and then there were like three more parts, and I was just like, actually, I can’t…I think it’s unrealistic to think that researchers (have time).” ID12

The most common structural issue was wanting more specific prompts, but also reorganising the layout was suggested by a few researchers.

“Almost like a list of the social graces, just to be like, you could think about class, you could think about geography, you could think about sexuality, just…or something like that.” ID5

“I found that I had to copy and paste the prompts just below each question so the way the form is designed at the moment it just has the top heading numbered with each question. I had to copy and paste the prompts just below the question to help me answer the question. Because I just found that they were very vague.” ID6

## Advisory group consultation

The Advisory Group comprised twelve public contributors from across the two locations. Several individuals self-identified as being from ethnic minority backgrounds and/or as having specific health conditions and disabilities. Some within the group had been involved as public contributors in previous research projects and a range of employment types and statuses were represented.

This consultation was undertaken because our emerging findings were suggesting that PCIE was the main way researchers would ensure inclusion of different ethnic groups. More information on the process and outputs of this consultation is provided in the supplementary material (Additional file 2). The main output of the Advisory Group is a guidance document that summarises the groups’ ‘Top tips’ for researchers to involve patient, public and community contributors in the INCLUDE Ethnicity Framework. The structure of this document and answers to questions were developed iteratively across sessions, and it was reviewed collectively within the groups as well as by individual group members, and therefore, it provides the most complete account of the findings. Here, we present a summary of the discussions.

### Initial meetings (Manchester and East Midlands separate groups): key questions

#### When and how to involve patient, public and community contributors in the INCLUDE Ethnicity Framework?

Members of the advisory group agreed that it was best to involve public and community contributors from the earliest stage possible.

However, people felt differently as to how they would want to be involved in the Ethnicity Framework. Some in the group expressed a preference for the Framework being completed by researchers before it was shared with an appropriate and diverse group of public contributors. Others said they would prefer the Framework to be completed together with the group, or on a one-to-one basis individually.

#### Who should make the first contact and how should this be made?

Ideally, the first contact was recommended to be through a known or trusted person. Community groups, and public involvement leads within these, and trusted healthcare professionals were seen to have a key role in this. For example, a GP/pharmacist was seen as a trusted professional by many.

Universities and clinical trial websites were not viewed as accessible due to a lack of awareness of their existence and some of the group said they would not always trust such organisations. There was a concern about who was funding university research and it is essential to make this clear.

Being proactive though, for example, outreach activities, was considered important because people are often busy and do not have time to visit lots of websites and look for studies or opportunities.

#### What should researchers say or ask about clinical trials?

One thing that was seen as helpful was allowing some time for patient, public and community contributors to say why they were there and specifically interested in inputting into this clinical trial.

A key issue identified was making sure everyone who could take part in a trial was able to. This included being clear about potential barriers but also respecting peoples’ choice not to take part if they did not wish to. For example, by definition, a trial is testing something out and this could be too anxiety-provoking for some people.

The group also highlighted that while it was important that any risks involved were properly explained, it was also important that benefits or incentives were discussed. Some of the group identified that it was important that researchers clearly point out the value and impact a trial could potentially have.

Another key consideration was allowing for cultural sensitivities when engaging with ethnic minorities or other under-served groups. Contributors reported the importance of doing so at all stages of the trial: when initially trying to recruit and once individuals from such groups are recruited into the trial.

### Meetings 2 and 3 (Manchester and East Midlands separate groups)

#### Why involve patient, public and community contributors in a specific trial?

Involving patient, public and community contributors will mean the decisions researchers make will be more considered, relevant, effective and sustainable.

Recommendations will then come from the people who may be participants in relevant trials and so will understand recruitment ‘on the ground’.

#### Why use this INCLUDE Ethnicity Framework?

PCIE groups should be informed (if they are not already aware) that ethnic minority and other under-served groups are under-represented in health research, including randomised trials.

The INCLUDE Ethnicity Framework is used to encourage researchers to think through the under-served groups relevant to their specific trial and to take appropriate action to improve engagement.

This may help researchers fund trials because they will have thought through their trial design in more detail and have gained invaluable insights regarding inclusion. Considering the full range of people you could recruit and how to do this could support recruitment during the trial.

#### Is the fact that the trial might not get funded an issue?

The INCLUDE Ethnicity Framework is ideally completed while a funding application for a trial is in development. Funding applications are often rejected and so the group was asked whether they thought this was an issue in terms of their continuous involvement and payment for time involved.

The group expressed that they would still prefer to be involved early and that researchers should make it clear that it was possible that the research would not be funded. Payment would still be needed, which can sometimes be provided by funders (e.g. researchers applying for NIHR funding can apply for funding to consult PCIE representatives when a funding application is in preparation).

### Final meeting (combined Manchester and East Midlands groups)

All the above questions were briefly reviewed and any final changes made to the guidance document. The guidance document is in Additional file 2.

## Discussion and conclusion

### Statement of principal findings

Through our three inter-related work packages, we were able to advance our understanding of the barriers research teams perceive when aiming to recruit and retain under-served groups within trials and what are the current approaches used. We also established researcher views on the usability and value of the INCLUDE Ethnicity Framework. We developed co-produced guidance to support future engagement using the Ethnicity Framework with PCIE representatives.

We found that most researchers recognised the importance of increasing engagement and recruitment of under-served groups within trials. However, there was considerable variation regarding whether researchers know how they might do this and whether they were proactively taking steps to increase the recruitment of such groups. Another key finding was that although researchers have a responsibility to employ more inclusive trial design and recruitment methods, there is an urgent need for shared resources and structural change to facilitate this. PPI/PCIE was identified as the most common method of trying to improve the recruitment of under-served groups to trials both within the survey and within the qualitative interviews. However, researchers also commonly identified that the PCIE groups for the trials they were discussing were not very diverse (the characteristics that researchers identified as of limited diversity were ethnicity, socio-economic status, full-time workers, age and gender identity). Furthermore, it is possible that if researchers felt more confident in the ways that they could conduct more inclusive research, then this would put less pressure on PCIE contributors and groups. This is not to negate the undoubted value of PCIE but rather indicates that other areas of research conduct and design also require careful consideration for research to be more inclusive [16]. The entire responsibility for inclusive research cannot be placed onto PCIE.

### Strengths and weaknesses of the study

A key weakness is that the qualitative study used convenience sampling and it is possible that respondents for both the survey and the qualitative study were researchers more committed to inclusive research (volunteer bias). This is especially likely to pertain to the qualitative interview (which took around 30min) as researchers were investing more time than in the brief survey. However, a large number of active researchers were sampled and the response rate for the survey was reasonable at 38%. Furthermore, within the participants who completed a qualitative interview, there was a range of experience and confidence (e.g. early career trial managers to experienced CIs with a special interest in diverse inclusion) in designing and undertaking inclusive research, as well as a range of trial contexts included. Another limitation is that two of the study team were involved in developing the INCLUDE Ethnicity Framework (ST, PB), which could have introduced bias. However, the interviewer and analyser (LM) for the qualitative aspect was not involved in developing the version of the INCLUDE Ethnicity Framework that was being evaluated and this may have made it easier for participants to provide critical feedback.

A key strength is that, to the authors’ knowledge, this is the only recent attempt to survey the current practice and perspectives of UK researchers on inclusive research (certainly since the introduction of the NIHR INCLUDE initiative; [18]). This study focuses on the UK context but the literature indicates that similar broad issues pertain in other contexts (e.g. [1]). Another strength is that this study examined the acceptability and usability of a tool to promote inclusive research (the Ethnicity Framework), thus adding to the relatively modest evidence base regarding what strategies can facilitate inclusive research.

### Meaning of the study

This study indicated that one important step in increasing recruitment and inclusion of under-served groups within trials was researcher awareness at the initial stage of trial design, for example, who might be excluded if the current trial design was not changed. This study indicates that the Ethnicity Framework is a tool that can facilitate such awareness. However, it needs to be included as part of early design discussions and is likely to need to be revisited once the trial is underway. A recent study, describing attempts to recruit an ethnically diverse sample to inform the development of an intervention for stroke patients, described the ways that even protocols that aim to recruit ethnic minority (and other minority groups) can pose barriers [16]: for example, the difficulty of junior, ethnic minority members of trial teams raising issues with more senior team members about trial design features that are likely to exclude individuals from ethnic minority groups [16]. These authors also highlight issues regarding ‘routinised methods and procedures that filter out participants who lack literacy, English fluency and digital proficiency, and research cultures and timelines that discourage the collection and analysis of data that might challenge the basic assumptions upon which a trial is built’. This indicates the complexity of negotiating ongoing inclusion within a trial and indicates a need for in-depth reflection, combined with the need for methodological resources to support inclusion that was also identified in the current study.

It was also clear from the study results that there were systemic barriers that were difficult and probably impossible for individual researchers to address and surmount (for example, structural inequalities within the NHS, such as discrimination and ethnic minority staff being under-represented at senior levels of the NHS [13, 19]). But it is important that this does not become more of a barrier than it already is. It is likely that while individuals from some under-served groups may be less likely to access services, such problems will be compounded if those who do access these services are not recruited into trials. Moreover, by being open about unequal use of current care provision, new more equitable forms of delivery may be developed. Similarly, lack of researcher time to complete the Ethnicity Framework was cited as a barrier. While not to underplay the challenges of workload, or the need for broader systemic change, there are important scientific and moral reasons for carefully considering how to involve people from under-served groups in a trial and these deliberations deserve time.

### Role of PCIE/PPI

Regarding the specific strategies identified by researchers as ways of improving involvement of under-served groups, PCIE was the most common approach used. Although PCIE is essential to understanding diverse perspectives, this study suggests that there is scope for researchers and funders to have a wider focus in terms of thinking about how PCIE can specifically support greater inclusion. In addition, PCIE groups in a range of research areas were described as not very diverse: an issue that has been identified elsewhere [6, 7, 14]. Furthermore, if this is the only strategy that is being employed, then involving diverse recruitment sites, properly resourced community outreach and other inclusion strategies that require time and funding may be less likely to be employed (as they may not have been included in original funding bids and timelines). It is also risks placing an overly high expectation and burden on the PCIE process and in turn PCIE contributors. While this was not the primary strategy used for all researchers (some used several), for others, it was the main or only strategy used.

Ways of facilitating involvement from diverse individuals within PCIE were suggested and discussed during the PCIE consultation process, such as making initial contact through a known or trusted person. Community groups, public involvement leads with good community links and trusted healthcare professionals were seen to have a key role in this. The group also highlighted that while it was important that any risks involved were properly explained, it was also important that benefits or incentives were discussed. Some of the group identified that it was important that researchers clearly point out the value and impact a trial could potentially have.

Discussions regarding this are occurring in other contexts. For example, recent NIHR training on ‘How to Incorporate Equality, Diversity and Inclusion (EDI) in Patient and Public Involvement (PPI)’ made several suggestions regarding increasing the diversity of PCIE groups, including advertising widely, building up community networks, work actively to empower people and break down the power differentials, multi-lingual resources and bilingual researchers. It is possible that similar strategies can be helpful for both more inclusive PCIE and more inclusive recruitment to trials, for example, engagement with community networks, considering and accommodating language needs, building trust and addressing power dynamics and multi-lingual researchers [1, 14].

One of the possible reasons why PCIE was so often described as a method to improve inclusion of under-served groups is the limited evidence and guidance regarding which strategies to increase engagement, recruitment and retention are the most effective. Also, participants identified that the extent to which PCIE requirements are built into funding bid and ethical approval processes was another reason why PCIE was a well-used strategy. There is an ongoing need for detailed and robust evidence that examines the use of different strategies to improve inclusion. However, even when detailed effectiveness data are not available, certain strategies are commonly used and experienced researchers within the current study (and other research contexts, e.g. [1]) described that using a range of such approaches was helpful, e.g. recruiting from sites where relevant under-served groups were prevalent, or using interpreters. Bodicoat and colleagues provide fifteen recommendations to increase inclusion based on a rapid review of the international published literature on the specific barriers in relation to inclusion in clinical trials, and evidence of approaches that have been effective in overcoming these. They also recommend the use of multiple strategies and ones tailored to each population’s particular circumstances, background and needs. However, even where potentially effective strategies were reported within the current study, researchers also identified difficulties in building in enough time and funding to enable under-served groups to be recruited within tight timelines and funding resources. These tight timeframes are generally set by the research team, but kept short in the belief that this is needed to make the bid competitive. Researchers would require support from funders to feel confident to extend timelines and request more funding beyond what they may have done in the past.

### Key recommendations

Clearly, more inclusive research requires changes at multiple levels and stages of the research process. For example, the NIHR is increasingly providing training in this area; for example, training linked to the INCLUDE resources [12]. Furthermore, regulatory agencies, such as The Food and Drug Administration (FDA), have recently produced guidance on steps that those looking to apply for a new drug application can take to increase the recruitment of under-served groups in their clinical trials [17].

Recommendations based on this study are as follows:

- Minor changes to the INCLUDE Ethnicity Framework. For example, adding more specific prompts, encouraging researchers to detail specific actions they could take, and providing additional resources (where available)
- Use the INCLUDE Ethnicity Framework and related resources when designing clinical trials, and then reviewing during the trial (e.g. once recruitment has commenced to ensure strategies are effective)
- More detailed and robust evidence that examines the use of different strategies to improve inclusion
- Researchers to consider the funding and time resources required for diverse and inclusive recruitment
- Funders to publicly signal that they support this time and resource when they are essential in the pursuit of better, more representative and generalisable research
- Consider diversity within PCIE groups and take proactive steps to involve relevant under-served groups

## Conclusions

Most researchers recognised the importance of increasing involvement of under-served groups within trials. Applicants generally considered the INCLUDE Ethnicity Framework as beneficial in raising awareness of the need to design trials in order to enable the inclusion of under-served groups. There is a need for researchers to consider the funding and time resources required for diverse and inclusive recruitment to trials and for funders to enable this by actively encouraging trial teams to build inclusive strategies into funding bids. Our data also indicate that it is important that researchers take proactive steps to involve relevant under-served groups in PCIE.

## Supplementary Information

**Additional file 1.** The participant flow diagram.

**Additional file 2.** Guidance for researchers to involve patient, public and community contributors in the INCLUDE Ethnicity Framework.

## Data Availability

The datasets during and/or analysed during the current study available from the corresponding author on reasonable request.
