# Supplementary material for: Evaluating a tool to improve engagement and recruitment of under-served groups in trials
Source: Trials. 2022 Oct 9;23:867. doi: 10.1186/s13063-022-06747-2 (PMC9549666; doi:10.1186/s13063-022-06747-2)
Supplement: Supplementary file 1 — Additional file 1. The participant flow diagram. [file 13063_2022_6747_MOESM1_ESM.pdf]

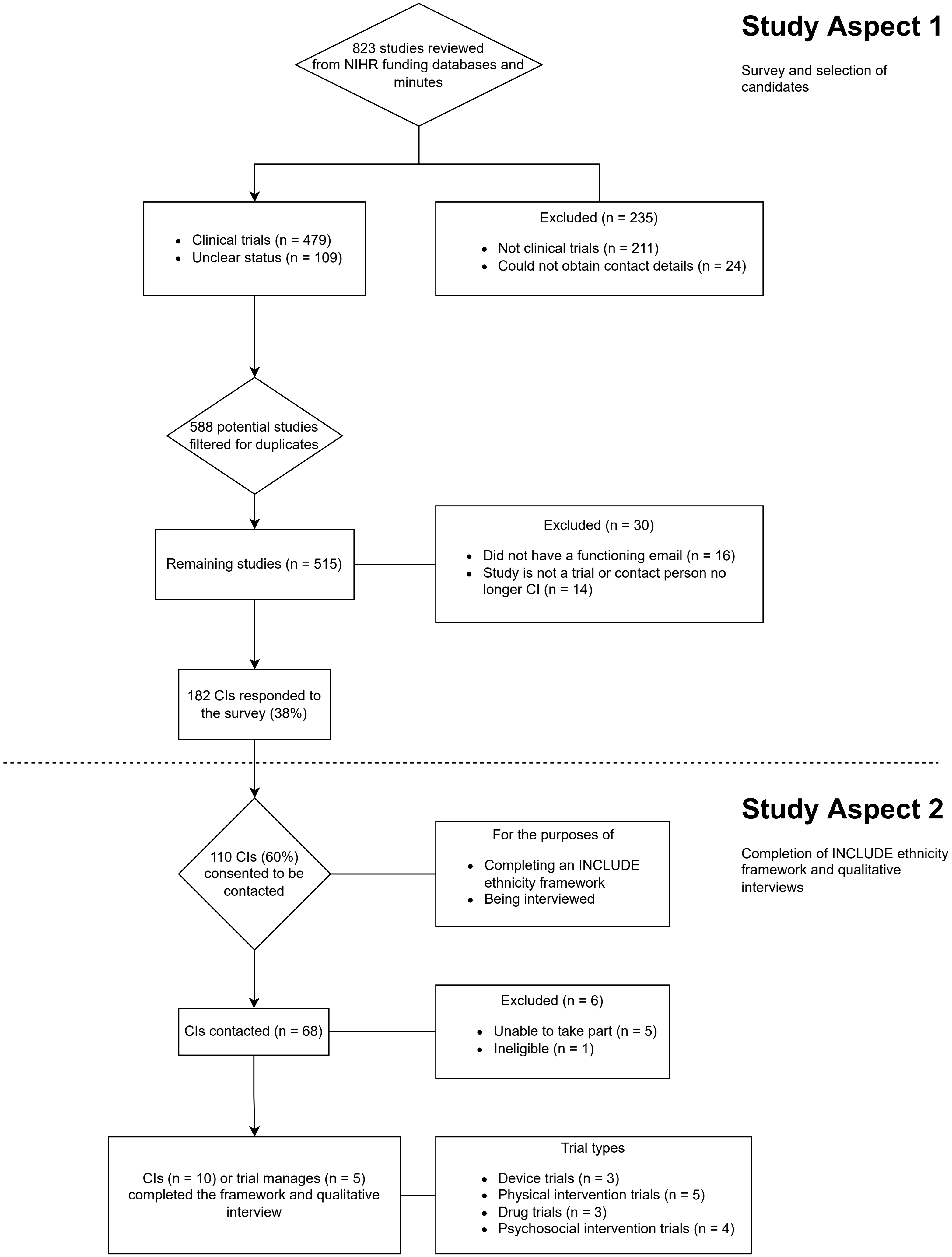
Study Aspect 1
Survey and selection of candidates
823 studies reviewed from NIHR funding databases and minutes
Clinical trials (n = 479)
Unclear status (n = 109)
Excluded (n = 235)
Not clinical trials (n = 211)
Could not obtain contact details (n = 24)
588 potential studies filtered for duplicates
Remaining studies (n = 515)
Excluded (n = 30)
Did not have a functioning email (n = 16)
Study is not a trial or contact person no longer CI (n = 14)
182 CIs responded to the survey (38%)
Study Aspect 2
Completion of INCLUDE ethnicity framework and qualitative interviews
110 CIs (60%) consented to be contacted
For the purposes of
Completing an INCLUDE ethnicity framework
Being interviewed
CIs contacted (n = 68)
Excluded (n = 6)
Unable to take part (n = 5)
Ineligible (n = 1)
CIs (n = 10) or trial manages (n = 5) completed the framework and qualitative interview
Trial types
Device trials (n = 3)
Physical intervention trials (n = 5)
Drug trials (n = 3)
Psychosocial intervention trials (n = 4)
