# Supplementary material for: Evaluating a tool to improve engagement and recruitment of under-served groups in trials
Source: Trials. 2022 Oct 9;23:867. doi: 10.1186/s13063-022-06747-2 (PMC9549666; doi:10.1186/s13063-022-06747-2)
Supplement: Supplementary file 2 — Additional file 2. Guidance for researchers to involve patient, public and community contributors in the INCLUDE Ethnicity Framework. [file 13063_2022_6747_MOESM2_ESM.docx]

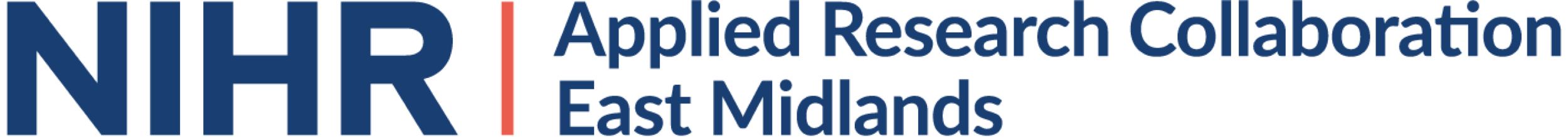

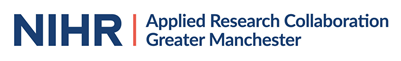


**Guidance for researchers to involve patient, public and community contributors in the INCLUDE Ethnicity Framework**

**Why involve patient, public and community contributors in a specific trial?**

- Involving patient, public and community contributors will mean the decisions you make will be more considered, relevant, effective and sustainable.
- Recommendations are coming from the people who may be participants in your trial and so will understand recruitment ‘on the ground’.

**Why use this INCLUDE Ethnicity Framework?**

- Ethnic minority and other under-served groups are under-represented in health research, including randomised trials.
- There are issues with how trials are designed that make it more likely that such groups will be excluded.
- The INCLUDE ethnicity framework encourages researchers to think through the under-served groups relevant to their specific trial and to take appropriate action to improve engagement.
- This may help you fund your trial because you will have thought through your trial design in more detail and have gained invaluable insights.
- Considering the full range of people you could recruit and how to do this could support recruitment during the trial.

**When and how to involve patient, public and community contributors in the INCLUDE Ethnicity Framework?**

- Members of the advisory group agreed that it was best to involve public and community contributors from the earliest stage possible. This will mean that researchers are more likely to design their trial incorporating the views, opinions, insights, needs and preferences of those it will potentially impact.
- However, people felt differently as to how they would want to be involved in the INCLUDE Ethnicity Framework. Some in the group expressed a preference for the Framework being completed by researchers before it was shared with an appropriate and diverse group of public contributors.
- However, others expressed they would prefer the Framework to be completed together with the group or on a one-to-one basis individually.
- If it was completed within a group of patient, community & public contributors, there were concerns regarding confidentiality.
- When the framework is completed within a group, it was requested that the language is straightforward and easy to understand. It was requested that researchers be mindful of technical / clinical language and if this was unavoidable then technical / clinical words and phrases should be properly explained so as to involve everyone. Training should be provided if needed.
- It was also noted that researchers often use longer sentences than necessary and this can make their point harder to understand.

**Who should make the first contact and how should this be made?**

- Ideally through a known or trusted person. Community groups, and public involvement leads within these, and trusted healthcare professionals were seen to have a key role in this. For example, a GP / pharmacist is seen as a trusted professional by many.
- Universities and Clinical Trial websites were not seen as accessible due to lack of awareness of their existence and some of the group said they would not always trust such organisations. There was a concern about who was funding university research and it is essential to make this clear.
- It was identified as important to identify unheard groups, reach out to people, engage in outreach activities, because people are often really busy and don’t have time to visit lots of websites and look for studies or opportunities.
- Clear information was seen as essential and this would help people decide if the research was well designed and likely to succeed if funded.
- It was highlighted how important that any risks involved were properly explained, but also that benefits or incentives were also explained clearly. Some of the group identified that it was important that researchers clearly pointed out the value and impact a trial could potentially have. For example, if it would help society in some way.

**What should researchers say or ask about clinical trials?**

- One thing that was seen as helpful was allowing some time for patient, public and community contributors to say why they were there and specifically interested in inputting into this clinical trial. It was recognised that often people get involved in advisory groups for projects that have a personal relevance for them.
- It was also identified that general questions about how people felt about a particular trial proposal would help to explore any positive features and highlight any potential barriers to taking part.
- A key issue identified was making sure everyone who could take part in a trial was able to. This included being clear about potential barriers but also respecting peoples’ choice not to take part if they did not wish to. For example, by definition a trial cannot have a 100% definite outcome, it is testing something out, and this could be too anxiety provoking for some people.
- But it was also recognised that if nobody took part in trials from ethnic minorities or other under-served groups then research would continue to be unrepresentative.
- It was requested that researchers were specific regarding who they were looking for, e.g. identifies as Indian or British Indian, rather than ‘South Asian’.
- Allow for cultural sensitivities when engaging with ethnic minorities or under-served groups. Do so at all stages of the trial: when initially trying to recruit and once individuals from such groups are recruited into the trial.

**Is the fact that the trial might not get funded an issue?**

- The INCLUDE ethnicity framework is ideally completed while a funding application for a trial is in development. Funding applications are often rejected and so the group was asked whether they thought this was an issue in terms of their continuous involvement and payment for time involved.
- The group expressed that they would still prefer to be involved early and that researchers should make it clear that it was possible that the research wouldn’t be funded. Several individuals within the group recognised that different funding bodies often had very specific criteria and this needed to be a key influence on how the research was framed.
- Working with organisations with a strong track record in successfully obtaining funding was also seen as important, as was funding for capacity building for charities and other organisations. It was recognised as helpful that if a bid was rejected then researchers should work in collaboration with Public Contributors to revise / strengthen the bid based on feedback received and then resubmit this to the same funding body or another funding body. Funding bodies can have different requirements. Researchers could also consider submitting related projects to different funders at the same time.
- It was also recognised that it can be helpful to have a network of studies on similar areas. So that if a study could not be funded then interested individuals could consult on other related projects.

**Developed by:** Patient and Public Involvement Groups based The University of Manchester and University of Leicester. A diverse group consisting of patients; carers and parents of all ages. Individual members were: Sumaira Naseem, Shofi Miah, Manoj Mistry, Russ Cowper, Kulsum Kabir, Nik Seth, Zeenat Chowdhury, Shah Jalal, Mohammed Asghar Yunus, Mohammed Abdus Shahid, Ilham Shah, Jackie Flynn. The groups acted as critical friends and they were very active participants in developing the document and project.

Supported by the National Institute for Health Research (NIHR) Applied Research Collaborations (ARCs) for Greater Manchester, and East Midlands. Groups were primarily coordinated and facilitated by Lydia Morris (University of Manchester, NIHR ARC Greater Manchester) and Nasima Miah (The Centre for Ethnic Health Research, NIHR ARC East Midlands).
